# Increased Experiences of Multiple Forms of Discrimination in Healthcare Settings During the COVID-19 Pandemic Among African, Caribbean, and Black (ACB) People Across Canada: A Cross-Sectional Survey

**DOI:** 10.3390/healthcare14101332

**Published:** 2026-05-13

**Authors:** Josephine Etowa, Amos Buh, Angela Kaida, Shamara Baidoobonso, Joseph Osuji, Judith Apondi Odhiambo, Lilian Ndongmo, Egbe Etowa, Bishwajit Ghose, David Este

**Affiliations:** 1 Faculty of Health Sciences, School of Nursing, University of Ottawa, Ottawa, ON K1S 5S9, Canada; lazan046@uottawa.ca; 2 School of International Development and Global Studies, University of Ottawa, Ottawa, ON K1N 6N5, Canada; 3 Faculty of Health Sciences, University of Buea, Buea P.O. Box 63, Southwest Region, Cameroon; 4 Faculty of Health Sciences, Simon Fraser University, Burnaby, BC V5A 1S6, Canada; angela_kaida@sfu.ca; 5 Department of Community Health and Epidemiology, Faculty of Medicine, Dalhousie University, Halifax, NS B3H 4R2, Canada; sbaidoob@outlook.com; 6 Faculty of Health and Community Studies, School of Nursing, Mount Royal University, Calgary, AB T3E 6K6, Canada; josuji@mtroyal.ca; 7 MAP Center for Urban Health Solution, Unity Health Toronto, Toronto, ON M5B 1W8, Canada; judith.odhiambo@mail.utoronto.ca; 8 Faculty of Community Services, Daphne Cockwell School of Nursing, Toronto Metropolitan University, Toronto, ON M5B 2K3, Canada; egbetowa@gmail.com; 9 Faculty of Health Sciences, Interdisciplinary School of Health Sciences, University of Ottawa, Ottawa, ON K1N 6N5, Canada; bghose@uottawa.ca; 10 Faculty of Social Work, University of Calgary, Calgary, AB T2N 1N4, Canada; deste@ucalgary.ca

**Keywords:** multiple discrimination, healthcare access, ACB people, COVID-19, Canada

## Abstract

**Background**: In Canada, racialized communities, including African, Caribbean, and Black (ACB) people, are disproportionately affected by HIV and COVID-19. Experiencing multiple forms of discrimination in healthcare settings compromises care engagement and health outcomes. The objective of this study was to assess the forms of discrimination ACB people experienced during the COVID-19 pandemic, discrimination levels experienced before and during the pandemic and the demographic factors associated with the increased experiences of discrimination among ACB people when accessing healthcare services during the pandemic. **Methods**: Data were collected via an online survey co-led by the Public Health Agency of Canada, University of Ottawa, ACB community leaders and researchers across Canada. Participants were recruited via email contact. To be eligible, a participant had to be living in Canada, be aged 18 years or older, be able to read English or French, and self-identify as an ACB individual. The survey captured information on access to health services and experiences of multiple forms of discrimination before and during the pandemic. We used multivariable logistic regression to identify factors associated with discrimination. **Results**: Of 1556 participants, 39.6% were aged 25–39, 42.7% were resident in Ontario, and 63.2% were of African origin. Prior to the COVID-19 pandemic, 62.1% reported having experienced at least one form of discrimination in a healthcare setting. During the COVID-19 pandemic, over 66% reported having experienced at least a form of discrimination, with 25% reporting a perceived increase in the frequency with which they experienced discrimination. The perceived increase in the frequency of discrimination was 10.8%, 15.3%, 15.9%, 17.0%, 18.1%, 18.7%, and 31.2% among participants who reported having experienced sexual orientation-, gender-, substance use-, disability-, age-, economic status-, and race-based discrimination, respectively. In the multivariate logistic regression, the odds of reporting increased experiences of discrimination in participants aged 50 and above were 0.38 times (95%CI: 0.21, 0.69) those in participants who were 31–40 years of age. **Conclusions**: The proportion of participants who reported an increased experience of discrimination during the pandemic was high. Although there is variation in levels of experienced discrimination, the different forms of discrimination (race-, gender-, sexual orientation-, substance use-, economic status-, disability- and age-based discrimination) that participants experienced are alarming. This underscores the need for concerted efforts to address multiple forms of discrimination in healthcare settings to improve care engagement and health equity among ACB communities. There was a significant association between perceived increased experience of discrimination and only one sociodemographic factor—older age (50 and above); other factors contributing to participants’ perceived increased experience of discrimination when accessing healthcare services need to be explored.

## 1. Background

The COVID-19 pandemic worsened health inequities and profoundly impacted healthcare systems worldwide [1,2]. It exacerbated existing health disparities and introduced new challenges especially among Black populations in Canada, who were disproportionately affected [3,4,5]. One critical area of concern is the experience of discrimination among this population within healthcare settings. This discrimination in healthcare manifested in various forms, including racial, ethnic, gender, and socioeconomic biases, which can significantly affect patient outcomes and healthcare access [5,6].

Prior to the pandemic, a prevalence of experiencing discrimination above 21% was reported in healthcare settings [7,8]. Racial and ethnic minorities, women, immigrants with precarious immigration statuses and individuals from lower socioeconomic backgrounds have been reported to have experienced biased treatments, which have contributed to disparities in health outcomes [9,10]. In fact, racial and ethnic minorities have frequently faced longer wait times, less thorough examinations, and lower quality of care compared to their White counterparts [11,12]. The most reported form of discrimination is race-based discrimination followed by discrimination based on education or income, weight, sex, disability including HIV, and age [13].

Since the start of the COVID-19 pandemic, healthcare systems have become overwhelmed, and the strain on resources have led to heightened stress, further resulting in burnout among healthcare workers [14,15]. The impact of this is profound on racial and ethnic minorities. In fact, a study found that racial and ethnic minorities were more likely to report negative healthcare experiences during the pandemic, which correlated with lower COVID-19 vaccination rates among these groups [3,16]. Additionally, women reported increased gender discrimination, including greater support needs and lower team cohesion [17].

Overall, forms of discrimination such as racial and ethnic discrimination experienced during the pandemic in healthcare settings have been documented in Canada and USA. Black, Indigenous, and other racialized individuals have worse healthcare service experiences compared to other racial and ethnic groups due to discrimination [13,18,19]. This discrimination led to lower rates of COVID-19 vaccination and increased difficulties in accessing healthcare, particularly among Asian, Indigenous, and Latino adults [16,20,21]. In fact, a study reported that 22.1% of participants experienced COVID-19-related discriminatory behaviours [22], and others stated that compared to White adults, people from all racial and ethnic minority groups were more likely to have experienced COVID-19-related discrimination [22,23,24]. In 2019, a national survey reported that for the past five years, discrimination was experienced by 33%, 44%, 24%, 29%, and 41% of Indigenous, First Nations, Metis, Inuit and Black people in Canada respectively [25]. Besides this, it has been documented that discrimination can occur on the basis of more than one perceived characteristic—an individual discriminated on grounds of ethnicity may also be discriminated on grounds of sexual orientation, gender, and or age, which creates cumulative disadvantage [26,27]. Furthermore, intersectionality approaches have reported that barriers to healthcare are not uniform and that racialized, low-income and immigrant people such as ACB people face intersecting oppressions that results in disproportionate risk of infection and mortality [28,29].

Given the intersecting nature of oppressions/types of discrimination, an analytical framework such as the intersectionality theory is needed to understand how overlapping and interconnected types of discrimination create severe vulnerabilities for specific populations such as ACB people [28]. In addition, for a comprehensive understanding of the cumulative burden of discrimination, and in order to develop targeted interventions and policies that recognize and address the unique experiences of individuals facing multiple discrimination, examining multiple forms of discrimination has been recommended [30,31]. Nevertheless, less is known about national patterns, perceived pandemic-related change, and multiple forms of discrimination measured together in the ACB population. The objective of this study therefore was to assess the various forms of discrimination experienced by ACB people during the pandemic, changes in the levels of discrimination experienced before and during the pandemic and the demographic factors associated with the increased experience of discrimination among ACB people when accessing healthcare services during the COVID-19 pandemic.

## 2. Methods

### 2.1. Study Design and Setting

This was a national, online, self-administered, cross-sectional survey to assess the impact of COVID-19 on access to sexually transmitted and blood-borne infection (STBBI)-related services among ACB people in Canada. The overall objective of the survey was to better understand the impact of the COVID-19 pandemic on access to STBBI-related services and also to assess experiences of discrimination when accessing services before and during the COVID-19 pandemic [32]. The survey design was inspired by the rapid assessment trendspotter methodology used in the European Monitoring Centre for Drug Addiction’s online survey on the impact of COVID-19. It was conducted online to avoid the COVID-19 risk associated with close contact with people when conducting face-to-face interviews. Further methodological details on this survey have been reported elsewhere [33].

### 2.2. Study Population, Participants, and Sampling

The study targeted ACB people who accessed healthcare services during the COVID-19 pandemic. To be eligible, a participant had to be living in Canada, be aged 18 years or older, be able to read English or French, and self-identify as an ACB individual.

Eligible participants were recruited through collaborative efforts between the Public Health Agency of Canada (PHAC), University of Ottawa, ACB community leaders and researchers across Canada. The survey link was distributed online through networks, community leaders, and stakeholders. The PHAC contacted over 800 stakeholders to participate in the study and to distribute the survey link to others. All stakeholders including service providers were encouraged to promote the survey link among their networks. Overall, more than 5000 organizations and individuals were emailed the survey link using the contact lists of stakeholders.

### 2.3. Data Collection

Data were collected through an online survey using Voxco. The questionnaire (Supplementary Materials) included items regarding health providers’ knowledge and experience working with ACB communities and their capacity to respond to the needs of ACB patients during and post-COVID-19. Data collection was conducted in both English and French with targeted efforts via key partner organizations at each site: Ottawa (Montfort Hospital, Ottawa Public Health and its affiliates—community health centres (CHCs), and long-term care facilities) and Toronto (St Mike’s Hospital, Taibu CHC; Women’s Health in Women’s Hands, etc.). Participant recruitment and data collection were conducted from 25 May 2021 to 12 July 2021.

Using a list of discrimination-related attributes, participants’ retrospective self-perceptions about the change in their experiences of discrimination when accessing healthcare services prior to and during the pandemic were asked. The response options included “increase”, “decrease”, “no change”, and “did not experience.” All participants that responded “increase”,” decrease” or “no change” to any discrimination attribute were considered to have experienced discrimination when accessing healthcare services.

### 2.4. Statistical Analysis

The collected data was analyzed using the statistical software programme STATA version 14. The participants’ sociodemographic characteristics (all categorical variables) have been described using frequencies and percentages.

To determine the levels of the various forms of discrimination experienced by participants, the proportion of participants who experienced any form of discrimination and the proportion of the perceived change in the frequency of discrimination experienced was computed. Since the percentage of non-responses to most variables was less than 10, this was considered as missing data and analyzed as such. The response options “increase”, “decrease”, and “no change” were considered as categories of each discrimination type and corresponding frequencies and percentages were computed.

To assess the sociodemographic factors associated with perceived increased experience of discrimination when accessing health services during the pandemic, bivariate and multivariate analyses were conducted. The bivariate analysis comprised using perceived increased experience of discrimination when accessing healthcare services as a binary outcome variable (coded as “increase” versus “decrease” and “no change”) and participants’ sociodemographic factors as correlates. The odds of perceived increased experience of discrimination between participants using unadjusted odds ratios, 95% confidence intervals and *p*-values were computed. A *p*-value ≤ 0.25 was set as the determining point in the bivariate analysis for a variable to be considered as appearing to have an association with perceived increased experience of discrimination and be included in the multivariate logistic model [34]. For the multivariate analysis, perceived increased experience of discrimination when accessing healthcare services during the pandemic was considered as a binary outcome variable and all variables with *p*-values ≤ 0.25 in the bivariate analysis were considered as correlates and included in the multivariate logistic regression model. Adjusted odds ratios, 95% confidence intervals and *p*-values were computed. All variables with *p*-values < 0.05 were considered to have a statistically significant association with perceived increased experience of discrimination when accessing healthcare services.

### 2.5. Ethical Considerations

The study was conducted in accordance with the Declaration of Helsinki, and the protocol was approved by the Health Canada and Public Health Agency of Canada (PHAC) Research Ethics Board (Project File Number: REB 2020-013P) on 29 October 2020, and an amendment to the protocol to include a qualitative component was approved by the Health Sciences and Science Research Ethics Board (REB) of the University of Ottawa (Ethics File Number: H-04-22-7687) on 5 May 2022. Written informed consent for participation and publication was obtained from all participants involved in the study.

## 3. Results

### 3.1. Participants’ Characteristics

The characteristics of the 1556 participants who took part in this study are presented in Table 1. Nearly 40% were between 25 and 39 years old. Participants were from all 10 provinces and three territories, with the largest proportion residing in Ontario (42.7%). Overall, 63.2% were Black Africans and 28.3% were Black Caribbeans. Two-thirds (66.2%) identified as cisgender women and the majority (81.8%) of participants were heterosexual or straight. Over a quarter of the participants (40.8%) were Canadian citizens, 29.0% of whom had been living in Canada for less than 5 years. Four hundred and twenty-nine (29.3%) participants had university graduate or professional degrees and about half of them (50.3%) were working full time. The majority of the participants (93.6%) had stable housing status with more than half of them having health insurance coverage.

### 3.2. Forms of Discrimination Experienced by Participants During the COVID-19 Pandemic

The forms of discrimination experienced by participants when accessing healthcare services during the COVID-19 pandemic are presented in Table 2. Of the 1556 participants included in this study, two-thirds (66.4%) of participants reported having experienced any form of discrimination during the pandemic. These participants who reported having experienced any discrimination further indicated the type they experienced. However, the reported experienced discrimination frequency varies by type.

(a) Race-based discrimination

Two-thirds (66.4%) reported having experienced race-based discrimination when accessing healthcare services during the pandemic. Of these, 31.2% reported having experienced an increase in race-based discrimination while 65.8% reported having experienced no change.

(b) Gender-based discrimination

More than half of the participants, 555 (57.8%), reported having experienced gender-based discrimination while accessing healthcare services during the pandemic. Although 81.6% reported having experienced no change in this discrimination frequency, 15.3% of participants reported having experienced an increase in the frequency with which they experienced gender-based discrimination when accessing healthcare services during the pandemic.

(c) Sexual orientation-based discrimination

Four hundred and forty-five (46.8%) participants reported having experienced sexual orientation-based discrimination when accessing healthcare services during the pandemic. Even though 86.3% of participants reported no change in the frequency with which this form of discrimination was experienced, 10.8% of participants reported having experienced an increase.

(d) Substance use-based discrimination

One-third of participants, 333 (35.5%), reported having experienced substance use-based discrimination. The majority of the participants (81.1%) did not report any change in the frequency of this discrimination. However, 53 (15.9%) participants reported having experienced an increase in this discrimination frequency during the pandemic.

(e) Economic status-based discrimination

More than half (57.3%) of participants reported having experienced economic status-based discrimination. While 376 (67.8%) did not report a change, 18.7% of participants reported having experienced an increase in this discrimination frequency.

(f) Disability-based discrimination

Three hundred and eighty-two (40.3%) participants reported experiencing disability status-based discrimination. Among this group, 77.0% reported no change in the frequency of this discrimination. Nevertheless, 17.0% of the participants reported having experienced an increase in this discrimination frequency.

(g) Age-based discrimination

Four hundred and sixty-three (48.8%) participants reported having experienced age-based discrimination when accessing healthcare services. Although many (80.4%) of these participants did not report any change in the frequency with which they experienced this form of discrimination, 18.1% reported an increase (Table 2).

### 3.3. Participants’ Discrimination Prior to and During COVID-19 Pandemic

**Figure 1:**
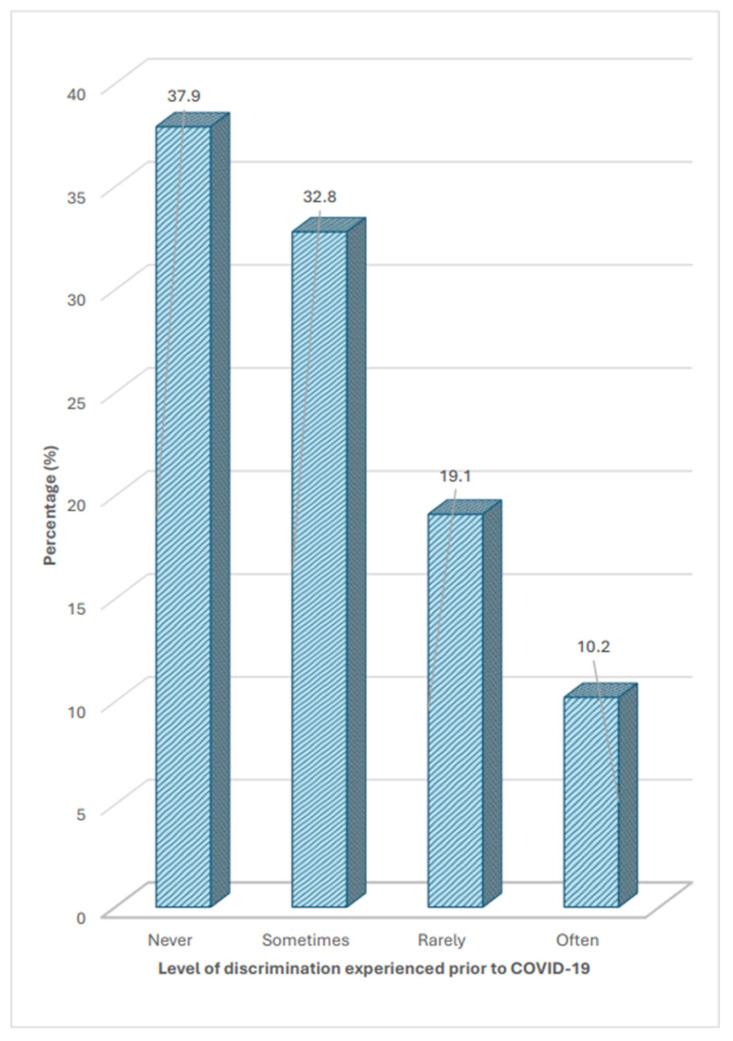
Proportion of participants and level of discrimination experienced prior to COVID-19 pandemic.

**Figure 2:**
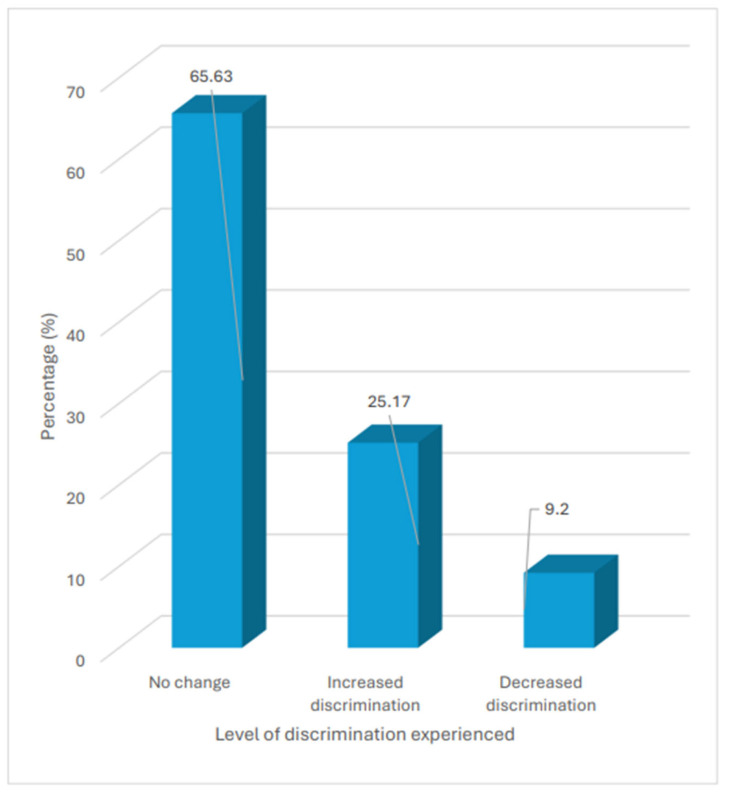
Proportion of participants and levels of discrimination experienced during COVID-19 pandemic.

Overall, only 37.9% of participants reported never experiencing any form of discrimination prior to the pandemic. Among those who reported having experienced any form of discrimination, 32.8% reported that these experiences only occurred sometimes (Figure 1). During the pandemic, although 65.6% of those who experienced discrimination did not report a change in the frequency with which they experienced it, 25.2% of them reported an increase in the frequency with which they experienced discrimination when accessing healthcare services (Figure 2).

### 3.4. Factors Associated with Increased Experiences of Discrimination During COVID-19

The factors associated with participants’ increased experience of discrimination during the pandemic are presented in Table 3 and Table 4. In the bivariate analysis, the factors that appeared to be associated with a participant’s increased experience of discrimination when accessing healthcare services during the pandemic included being a resident of the northern provinces or territories when compared to being a resident of the western province, being aged above 50 when compared to ages 31–40, having a heterosexual sexual orientation when compared to non-heterosexual sexual orientation, being a Canadian citizen compared to non-Canadian citizens, living in Canada for more than 10 years compared to living in Canada for less than 5 years, having some college/vocational training education compared to those with high school level of education, and living with family when compared to living with a friend/roommate. In fact, the odds of increased experiences of discrimination in participants resident in the northern provinces or territories were 0.52 times (95%CI: 0.19, 1.47) those in participants resident in the western province. The odds of increased experiences of discrimination comparing participants aged 50 and above to participants 31–40 years of age were 0.43 times (95%CI: 0.27, 0.68). Also, the odds of increased experiences of discrimination in heterosexual or straight sexual orientation participants were 0.79 times (95%CI: 0.54, 1.16) those in non-heterosexual sexual orientation participants. The odds of increased experiences of discrimination in participants with Canadian citizenship were 0.72 times (95%CI: 0.51, 1.03) those in participants who were non-Canadian citizens. With regards to the number of years lived in Canada, the odds of increased experiences of discrimination in participants who had been living in Canada for more than 10 years were 0.72 times (95%CI: 0.47, 1.13) those in participants who had been living in Canada for less than 5 years. Furthermore, the odds of increased experiences of discrimination in participants with some college/vocational training education were 0.75 times (95%CI: 0.50, 1.11) those in participants who had attained high school level of education. When considering participants’ housing status, the odds of increased experiences of discrimination in participants living with family were 0.75 times (95%CI: 0.49, 1.15) those in participants living with a friend/roommate (Table 3).

After adjusting for potential confounding by each of the demographic factors that appeared to have an association with the experience of discrimination by a participant when accessing healthcare services during the COVID-19 pandemic, only being of an older age (50 and above) remained a significant factor associated with experiencing discrimination. The odds of increased experiences of discrimination in participants aged 50 and above were 0.38 times (95%CI: 0.21, 0.69) those in participants who were 31–40 years of age (Table 4).

## 4. Discussion

In this study, we assessed the levels of discrimination experienced by ACB people when accessing healthcare services before and during the COVID-19 pandemic, the various forms of discrimination they experienced, and the factors associated with their increased experiences of discrimination during the COVID-19 pandemic. We document that prior to the COVID-19 pandemic, more than a quarter of the participants reported that they never experienced any form of discrimination. For those that experienced at least one form of discrimination, approximately 33% of them reported experiencing it sometimes. However, we also document that since the start of the pandemic, over 66% of participants experienced at least a form of discrimination with more than 25% of them reporting a perceived increase in the frequency with which they experienced discrimination when accessing healthcare services. Added to these, we document a perceived increase in the frequency with which participants experienced various forms of discrimination when accessing healthcare services—31.2%, 15.3%, 10.8%, 15.9%, 18.7%, 17.0%, and 18.1% of participants reported a perceived increase in their experience of race-, gender-, sexual orientation-, substance use-, economic status-, disability-, and age-based discrimination respectively. In this study, available participants’ sociodemographic factors did not accurately distinguish between participants who perceived an increased experience of discrimination and those who did not when accessing healthcare services during the COVID-19 pandemic. However, being of an older age (50 and above) had a statistically significant association with the participant’s perceived increased experience of discrimination when accessing healthcare services during the pandemic. Many studies have consistently reported that advanced age is a significant, independent risk factor for experiencing discrimination—in the UK and USA in particular, studies indicate that older adults aged 50 or 55 and older report frequent experiences of unfair treatment [35,36,37]. In Canada, it has also been documented that people over 50 years old who are living in Canada’s Northen Territories face higher risk of discrimination [13,38]. Potential reasons for this might be due to intersecting factors such as a convergence of systemic ageism, extreme geographic isolation, limited infrastructure, and the compounding effects of cultural, economic, and health disparities which make this demographic particularly vulnerable to both overt and systemic discrimination [13,39].

While the overall level of perceived increased experience of discrimination during the pandemic appears high (25%) in this study, it is within the range of levels of discrimination reported in similar studies conducted elsewhere. We found no studies that have assessed changes in participants’ experienced levels of discrimination prior to and during the pandemic in Canada before this study, but the levels of discrimination participants have experienced when accessing healthcare services ranges from 3.5% to 64.5% [7,8,16,18,22,25,40,41,42,43,44]. The highest level of discrimination (64.5%) reported so far was experienced by participants in a study conducted in the USA [43].

A series of studies have reported on the various forms of discrimination experienced by ACB people including discrimination based on race/ethnicity, gender discrimination, and sexual orientation discrimination [8,22,40,45,46,47,48]. Others have reported that the racial/ethnicity-based form of discrimination is very common [8,13,22,23,43]. In our study, however, in addition to the various forms of discrimination ACB people experienced while accessing healthcare, we also report the frequency with which participants perceived an increased experience of the forms of discrimination.

Some studies have found that factors significantly associated with participants’ experience of discrimination include age, language, education, income and place of residence [8,22,42,49]. While our study sample was adequate for estimating the levels of discrimination experienced by participants when accessing healthcare services during the COVID-19 pandemic, only one covariate (age) had a statistically significant association with the increased experience of discrimination. This is similar to studies that found that age had a statistical association with discrimination [8,42,49], but dissimilar to studies that found loneliness, depression, level of education, income level and place of residence as factors associated with discrimination [22,43]. The implications of experiencing discrimination on healthcare access and health outcomes are very negative. On healthcare access, the impact of experiencing discrimination causes people to delay or avoid care, experience unequal treatment, and to distrust the healthcare system [50,51,52]. On health outcomes, the experience of discrimination leads to worse health outcomes such as high rates of chronic diseases, poor mental health, increased hospital waiting times and worse outcomes for certain conditions like diabetes [11,43,53]. Furthermore, the experience of discrimination also has profound policy and structural implications, shifting from individual acts of prejudice to deeply embedded systemic issues affecting organizational culture, legal frameworks, and societal equity [54,55]. Structurally, discrimination creates systemic inequity, institutional barriers, segregation and disinvestment, economic disparity and institutional mistrust [56]. Policy-wise, the implications of discrimination are extensive, resulting in the need for legal, organizational, and societal frameworks that ensure equality, protect human rights, and prevent systemic disadvantages. Comprehensive strategies such as anti-harassment policies, duty-to-accommodate procedures, and proactive measures to remove barriers are required to fight discrimination [57,58].

## 5. Limitations and Strengths of This Study

The levels of the various forms of discrimination reported in this study could be influenced by population characteristics and the policies that were enforced to fight the COVID-19 pandemic which might have either limited access to healthcare services or affected participants’ perception of how they were treated when accessing healthcare services. Although this was a national survey, the sample may not have been representative of all ACB people who faced discrimination when accessing healthcare services during the pandemic. Also, the study participants are ACB people who sought access to sexually transmitted and blood-borne infection-related services; this sample might have been facing discrimination that may not be at the same level as other ACB people who sought other medical services. Furthermore, the study only included a cross-section of ACB people who could fill out the survey online—thus, digital access disparities, language limitations beyond English and French, and potential social desirability bias in reporting discrimination experiences might limit the representation of our study sample. The levels of the various forms of discrimination reported therefore reflect only the detection of cases of experienced discrimination in our online survey and not necessarily the incidence of discrimination or the overall level of experienced discrimination in the Canadian ACB population. Participants’ differences in use of online services might also have contributed to an underestimation of the proportion of participants that experienced discrimination but may not have been able to respond to our online survey. Also, the study employed non-probability sampling and measured retrospective perceptions of change in experiences of discrimination, rather than observed change over time; there is therefore the possibility of response/selection bias as participants might not have revealed the truth about their experiences during the pandemic. Equally, there might be recall bias as participants might not have fully remembered whether they experienced discrimination or not. Missing data related especially to some participants’ characteristics and responses not included in the analysis reduced sample sizes in some discrimination categories and thus affected the accurate calculation of sampling variability.

Nevertheless, this study was strengthened by the fact that it was a national survey that was available online, making it possible for ACB people across the country to participate in the study. In addition, the online survey enabled data collection from many participants within a short time. Our statistical analysis also allowed for some description of the levels of the various forms of discrimination participants experienced when accessing healthcare services during the pandemic and the factors associated with the experience of discrimination regarding ACB people in Canada.

## 6. Conclusions

The proportion of participants with perceived increased experience of discrimination when accessing healthcare services during the COVID-19 pandemic was high. Although there is variation in levels of experienced discrimination, the different forms of discrimination participants experienced are alarming. This underscores the need for concerted efforts to address multiple forms of discrimination in healthcare settings in order to improve care engagement and health equity among ACB communities. There was a significant association between perceived increased experience of discrimination and only one sociodemographic factor—older age (50 and above); other factors contributing to participants’ perceived increased experience of discrimination when accessing healthcare services need to be explored. Future studies including both online surveys and face-to-face interviews with ACB people are needed to assess trends in experienced discrimination and related determinants. 

## Figures and Tables

**Table 1:** Characteristics of the overall study participants.

Characteristic	N	%
**Province or territory of residence**		
British Columbia	169	10.9
Alberta	170	10.9
Saskatchewan	30	1.9
Manitoba	19	1.2
Ontario	664	42.7
Quebec	197	12.7
New Brunswick	62	4.0
Nova Scotia	54	3.5
Prince Edward Island	67	4.3
Newfoundland and Labrador	81	5.2
Territories	39	2.5
None of the above	2	0.1
**Age (years)**		
18–24	178	11.4
25–39	616	39.6
40–54	519	33.4
55–64	173	11.1
65+	70	4.5
**Race or racial background**		
Black African	983	63.2
Black Caribbean	441	28.3
Black Indigenous or Black Canadian	113	7.3
Black American	27	1.7
Black Latin American	17	1.1
Multiracial (one Black parent)	103	6.6
Another Black race	25	1.6
**Gender identity**		
Cisgender woman	990	66.2
Cisgender man	462	30.9
Transfeminine	11	0.7
Transmasculine	33	2.2
**Sexual orientation**		
Heterosexual or straight	1271	81.8
Gay or lesbian	73	4.7
Bisexual	102	6.6
Two-spirit	10	0.6
Other	55	3.5
Don’t know	42	2.7
**Citizenship status**		
Canadian-born citizen	358	23.2
Canadian-naturalized citizen	630	40.8
Permanent resident	291	18.8
Refugee or protected person	59	3.8
Refugee claimant or person needing protection	35	2.3
Asylum seeker	5	0.3
Temporary resident	155	10.0
Undocumented or no immigration status	7	0.5
Other	6	0.4
**Number of years lived in Canada (years)**		
<5	282	29.0
5–9	229	23.5
10–14	167	17.2
15–19	81	8.3
20–24	63	6.5
25+	152	15.6
**Education**		
Less than high school	67	4.6
High school	105	7.2
Some college/vocational training	94	6.4
Completed college/vocational training	159	10.9
Some university	111	7.6
Completed university certificate or diploma	131	9.0
University undergraduate degree	356	24.4
University graduate or professional degree	429	29.3
Other	10	0.7
**Employment**		
Unemployed	336	22.6
Student	141	9.5
Work part time	263	17.7
Work full time	749	50.3
**Housing status**		
Stable housing	1456	93.6
Precarious housing	99	6.4
**Health insurance coverage**		
Yes	1031	66.3
No	373	24.0
Don’t know	150	9.7

N = frequency, % = frequency in percentage.

**Table 2:** Forms of discrimination experienced by participants when accessing healthcare services.

Indicator	N	%
**Experienced race-based discrimination since start of COVID-19 pandemic**		
None	328	33.6
Any	647	66.4
**Change in experienced race-based discrimination since start of pandemic**		
Increase	202	31.2
No change	426	65.8
Decrease	19	2.9
**Experienced gender-based discrimination**		
None	405	42.2
Any	555	57.8
**Change in experienced gender-based discrimination**		
Increase	85	15.3
No change	453	81.6
Decrease	17	3.1
**Experienced sexual orientation-based discrimination**		
None	505	53.2
Any	445	46.8
**Change in experienced sexual orientation-based discrimination**		
Increase	48	10.8
No change	384	86.3
Decrease	13	2.9
**Experienced substance use-based discrimination**		
None	604	64.5
Any	333	35.5
**Change in experienced substance use-based discrimination**		
Increase	53	15.9
No change	270	81.1
Decrease	10	3.0
**Experienced economic status-based discrimination**		
None	414	42.7
Any	555	57.3
**Change in experienced economic status-based discrimination**		
Increase	104	18.7
No change	376	67.8
Decrease	75	13.5
**Experienced disability-based discrimination**		
None	565	59.7
Any	382	40.3
**Change in experienced disability-based discrimination**		
Increase	65	17.0
No change	294	77.0
Decrease	23	6.0
**Experienced age-based discrimination**		
None	484	51.1
Any	463	48.9
**Change in experienced age-based discrimination**		
Increased	84	18.1
No change	372	80.4
Decreased	7	1.5

N = frequency, % = frequency in percentage.

**Table 3:** Factors associated with increased experience of discrimination during COVID-19 pandemic—bivariate analysis.

Characteristic	N	%	Increased Experience of Discrimination		*p*-Values
			OR	95%CI	
**Province or territory of residence**					
Western (BC)	36	27.69	Ref		
Central (ON, QC)	27	25.47	0.89	0.50–1.60	0.70
Prairies (AB, SK, MB)	112	24.14	0.83	0.54–1.29	0.41
Atlantic (NB, NS, PEI, NL)	47	27.33	0.98	0.59–1.63	0.94
Northern (YT, NWT, NU)	5	16.67	0.52	0.19–1.47	0.22
**Age (years)**					
31–40	79	30.74	Ref		
≤20	4	14.81	0.39	0.13–1.17	0.09
21–30	54	25.23	0.76	0.51–1.14	0.19
41–50	58	28.43	0.90	0.60–1.34	0.59
>50	32	16.00	0.43	0.27–0.68	<0.01
**Gender identity**					
Cisgender female or transfeminine	153	25.21	Ref		
Cisgender male or transmasculine	66	24.00	0.94	0.67–1.31	0.70
**Sexual orientation**					
Not heterosexual or straight	45	29.03	Ref		
Heterosexual or straight	182	24.43	0.79	0.54–1.16	0.23
**Canadian citizen**					
No	85	27.96	Ref		
Yes	81	21.95	0.72	0.51–1.03	0.07
**Number of years lived in Canada (years)**					
<5	48	31.17	Ref		
5–10	42	22.46	0.64	0.39–1.04	0.07
>10	64	24.71	0.72	0.47–1.13	0.15
**Education**					
High school	61	28.77	Ref		
Less than high school	22	24.18	0.79	0.45–1.39	0.41
Some college/vocational training	71	23.20	0.75	0.50–1.11	0.15
Completed university certificate or diploma	72	25.71	0.86	0.57–1.28	0.45
**Housing status**					
Living with friend/roommate	38	29.23	Ref		
Living with family	140	23.73	0.75	0.49–1.15	0.19
Living alone/shelter/homeless	48	28.57	0.97	0.58–1.60	0.90
**Employment**					
Unemployed	42	26.75	Ref		
Student	18	24.66	0.90	0.47–1.70	0.74
Work part time	43	26.38	0.98	0.60–1.61	0.94
Work full time	120	24.19	0.87	0.58–1.32	0.52

OR = unadjusted odds ratio, Ref = reference variable category, CI = confidence interval, *p*-value < 0.25 suggests possible association with increased experiences of discrimination.

**Table 4:** Factors associated with increased experience of discrimination during COVID-19 pandemic—multivariable analysis.

Characteristic	N	%	Increased Experience of Discrimination		*p*-Values
			aOR	95%CI	
**Province or territory of residence**					
Western (BC)	36	27.69	Ref		
Central (ON, QC)	27	25.47	0.89	0.43–1.85	0.75
Prairies (AB, SK, MB)	112	24.14	0.83	0.48–1.44	0.52
Atlantic (NB, NS, PEI, NL)	47	27.33	0.74	0.37–1.47	0.39
Northern (YT, NWT, NU)	5	16.67	0.41	0.11–1.57	0.19
**Age (years)**					
31–40	79	30.74	Ref		
≤20	4	14.81	0.77	0.21–2.81	o.69
21–30	54	25.23	0.65	0.37–1.13	0.13
41–50	58	28.43	0.74	0.37–1.47	0.39
>50	32	16.00	0.38	0.21–0.69	<0.01
**Sexual orientation**					
Not heterosexual or straight	45	29.03	Ref		
Heterosexual or straight	182	24.43	0.78	0.45–1.33	0.36
**Canadian citizen**					
No	85	27.96	Ref		
Yes	81	21.95	0.75	0.41–1.35	0.34
**Number of years lived in Canada (years)**					
<5	48	31.17	Ref		
5–10	42	22.46	0.75	0.41–1.31	0.31
>10	64	24.71	1.11	0.56–2.22	0.77
**Education**					
High school	61	28.77	Ref		
Less than high school	22	24.18	0.82	0.37–1.83	0.63
Some college/vocational training	71	23.20	0.72	0.42–1.23	0.23
Completed university certificate or diploma	72	25.71	0.90	0.52–1.56	0.71
**Housing status**					
Living with friend/roommate	38	29.23	Ref		
Living with family	140	23.73	0.83	0.47–1.45	0.51
Living alone/shelter/homeless	48	28.57	1.35	0.69–2.66	0.39

aOR = adjusted odds ratio, Ref = reference variable category, CI = confidence interval, *p*-values < 0.05 are statistically significant association with increased experiences of discrimination.

## Data Availability

The data presented in this study are available on request from the corresponding author due to ongoing analysis of the data—the study involved both healthcare providers and ACB people and the analysis of the healthcare provider component is still ongoing; we will release the data after this analysis.

## Supplementary Materials

The following supporting information can be downloaded at: https://www.mdpi.com/article/10.3390/healthcare14101332/s1, Survey of the impact of COVID-19 on access to STBBI and related health services in African, Caribbean and Black communities in Canada.

## Abbreviations

The following abbreviations are used in this manuscript:

AB	Alberta
ACB	African, Caribbean and Black
aOR	Adjusted odds ratio
BC	British Columbia
CHC	Community health centre
COVID-19	Coronavirus disease 2019
CI	Confidence interval
HIV	Human immunodeficiency virus
MB	Manitoba
N	Frequency
NB	New Brunswick
NL	Newfoundland and Labrador
NS	Nova Scotia
NU	Nunavut
NWT	Northwest Territories
ON	Ontario
OR	Odds ratio
PEI	Prince Edward Island
PHAC	Public Health Agency of Canada
QC	Quebec
REB	Research Ethics Board
Ref	Reference variable
SK	Saskatchewan
STBBI	Sexually transmitted and blood-borne infection
USA	United States of America
YT	Yukon Territories
