# Supplementary material for: Increased Experiences of Multiple Forms of Discrimination in Healthcare Settings During the COVID-19 Pandemic Among African, Caribbean, and Black (ACB) People Across Canada: A Cross-Sectional Survey
Source: Healthcare (Basel). 2026 May 13;14(10):1332. doi: 10.3390/healthcare14101332 (PMC13206055; doi:10.3390/healthcare14101332)
Supplement: Supplementary file 1 [file healthcare-14-01332-s001.zip › healthcare-4167602-supplementary.pdf]

Survey of the impact of COVID-19 on access to STBBI and related health services in African, Caribbean and Black communities in Canada

The Public Health Agency of Canada wants your help to better understand how the COVID-19 pandemic has affected access to services for the care and prevention of HIV, hepatitis C and other sexually transmitted and blood-borne infections (STBBI). We also want to know how access to drug treatment and harm reduction services (such as addictions counselling and needle distribution programs) have been affected.

What is this survey about?
This survey asks questions about changes in access to these services due to the COVID-19 pandemic. It also asks about mental health, racism, stigma and discrimination, substance use, domestic violence and your housing, social and economic situation because these issues have an impact on access to services.

Who can participate in this survey?
Some groups in Canada, including African, Caribbean and Black communities, are more affected by STBBI than other people in Canada. If you identify as a member of African, Caribbean and Black communities and are 18 years or older, you are eligible to participate in this survey.

The survey is entirely voluntary. No directly identifying personal information such as your name or phone number is asked. The information you provide will be kept strictly confidential. You can stop completing the survey at any time.

Your participation is important!
Your participation is important so that the information collected is as accurate and complete as possible. This information will be used by the Public Health Agency of Canada as well as a research group that has been contracted by the Public Health Agency of Canada based out of the University of Ottawa that focuses on the health of African, Caribbean and Black communities in Canada. This information will help evaluate the need for appropriate support measures, during and after the pandemic and will not be used or shared for any other purpose. Only information about groups of people will be shared with public health organizations across Canada to identify changes in the delivery of these health services.

What about privacy of my personal information?
The personal information you provide to the Public Health Agency of Canada is being collected under section 4 of the *Department of Health Act* and section 3 of the *Public Health Agency of Canada Act* and is governed in accordance with the *Privacy Act*. It will only be used for the purpose outlined in this notice. Information from partially completed or unsaved questionnaires may also be retained and used.

What are my rights regarding collection of personal information in this survey?
You have a right to contact the Privacy Commissioner of Canada if you feel your personal information has been handled improperly. For more information about these rights, or about how we handle your personal information, please contact the survey team at: phac.tracks.aspc@canada.ca.

How will the results be made available?
Survey findings will be available in public health reports and other public documents and will always refer to groups of people, never to one person.

How long does it take to complete the survey?
It will take about 10-20 minutes to complete the survey.

What if I find any of the questions upsetting?
If you think you need mental health support after completing the survey, help and resources are available to you at this website: Mental health support: Get help

Need more information about this survey?
For more information about the survey contact the survey team at: phac.tracks.aspc@canada.ca

This online survey will be open from May 25, 2021 to June 25, 2021. Please participate now!

By clicking the *Start Survey* button, you have read and understood the information on this page and consent to participation.

Start Survey

Important features to navigate the survey

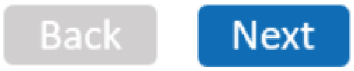

Use the **Back** and **Next** buttons located at the bottom of the page.
Do not use the navigation buttons at the top of your browser or the corresponding shortcut keys.
Based on your answers to certain questions, the survey will automatically skip any questions that do not apply to your situation.

Session timeout

After **2 hours of inactivity**, your session will time out.

Start of Survey

Note: Participants are prompted to complete each question should they miss or skip an answer. Should they prefer not to answer any question, they can hit 'Next' to go to the next question. In this instance, REFUSED will be recorded as their response.

1.1 How old are you? _____
[Min = 0; Max = 150] → If under 18 years skip to Error Message 1b

1.2 Are you currently in Canada? *Check one only.*

- ☐ Yes
- ☐ No → Skip to Error Message 1b

1.3 People living in Canada come from many different cultural and racial backgrounds. Do you consider yourself to be any of the following? *Select all that apply.*

- ☐ Black African
- ☐ Black Caribbean
- ☐ Black Indigenous or Black Canadian
- ☐ Black American
- ☐ Black Latin American
- ☐ Multiracial (where one parent is Black)
- ☐ Another Black race
- ☐ None of the above

→ If under 18 years, live outside of Canada, skip to Error Message 1b
→ For ACB survey, if 1.3=only None of the above, skip to Error Message 1b

Error Message 1b: You are not eligible to participate. Thank you for your time and interest in this survey.

1.4 What was your sex assigned at birth, meaning on your original birth certificate? We are asking this question so that we can use the information we get from this survey to better inform services for trans people. *Check one only.*

- ☐ Male
- ☐ Female
- ☐ Don't know
- ☐ Prefer not to answer
- ☐ REFUSED (option is hidden on Voxco)

1.5 Do you identify as:

If you have lived experience as trans, a history of gender transition, or are transgender, please select the gender you identify as. We recognize that gender identity questions are imperfect. Please select the option that fits best at this time. *Check one only.*

- ☐ Male } *→ If the responses from 1.4 and 1.5 do not match, go to question 1.6*
- ☐ Female } *→ If they do match, go to question 1.7*
- ☐ Two-spirit or other cultural gender identity *→ Go to question 1.6*
- ☐ Other, like gender fluid or non-binary *→Go to question 1.6*
- ☐ Don't know
- ☐ REFUSED (option is hidden on Voxco)

1.6 What gender do you currently live as in your day-to-day life? *Check one only.*

- ☐ Male
- ☐ Female
- ☐ Sometimes male, sometimes female
- ☐ Other / not listed
- ☐ Don't know
- ☐ REFUSED (option is hidden on Voxco)

1.7 Which of the following best describes your sexual orientation? *Check one only.*

- ☐ Heterosexual or straight
- ☐ Gay or lesbian
- ☐ Bisexual
- ☐ Two-spirit
- ☐ Other
- ☐ Don't know
- ☐ REFUSED (option is hidden on Voxco)

1.8 Where do you currently live? *Check one only.*

- ☐ British Columbia
- ☐ Alberta
- ☐ Saskatchewan
- ☐ Manitoba
- ☐ Ontario
- ☐ Quebec
- ☐ New Brunswick
- ☐ Nova Scotia
- ☐ Prince Edward Island
- ☐ Newfoundland and Labrador
- ☐ Yukon
- ☐ Northwest Territories
- ☐ Nunavut
- ☐ None of the above
- ☐ Don't know
- ☐ REFUSED (option is hidden on Voxco)

1.9 What are the first three characters of your postal code? _____ _____ ______

Example: A9A

☐ Don't know
☐ REFUSED (option is hidden on Voxco)

1.10 Where were you born? *Check one only.*

☐ Born in Canada → *Go to question 1.14*
☐ Born outside Canada
☐ REFUSED (option is hidden on Voxco)

1.11 Are you a Canadian citizen? *Check one only.*

☐ Yes → *Go to question 1.13*
☐ No
☐ REFUSED (option is hidden on Voxco)

1.12 What is your current residency or immigration status in Canada? *Check one only.*

☐ Landed immigrant or permanent resident
☐ Convention refugee or protected person
i.e., you have been formally approved as a refugee
☐ Refugee claimant or person in need of protection
i.e., you have applied to become a refugee but your application has not been approved yet
☐ Asylum seeker
i.e.,you are a person seeking refugee status but have not yet been processed
☐ Temporary resident
e.g., student, temporary worker, visitor, super visa (parent and grandparent)
☐ Undocumented or no immigration status
☐ Other
☐ Don't know
☐ Prefer not to answer
☐ REFUSED (option is hidden on Voxco)

1.13 How long have you lived in Canada? *Check one only.*

☐ Number of years: _______ (round up to the nearest year)
☐ Less than one year
☐ Prefer not to answer
☐ REFUSED (option is hidden on Voxco)

1.14 What is your highest level of education or training? *Check one only.*

- ☐ No formal schooling
- ☐ Less than high school
- ☐ Some high school
- ☐ Completed high school
- ☐ Some college, CEGEP, vocational school, trade school, or apprenticeship training
- ☐ Completed college, CEGEP, vocational school, trade school, or apprenticeship training
- ☐ Some university
- ☐ Completed university certificate or diploma
  e.g., Certificate in Project Management, Human Resources, etc.
- ☐ Completed undergraduate university degree
- ☐ Completed graduate or professional university degree
- ☐ Other
- ☐ Don't know
- ☐ Prefer not to answer
- ☐ REFUSED (option is hidden on Voxco)

1.15 Since the start of the COVID-19 pandemic, do you have insurance that covers all or part of your healthcare costs and/or prescription drugs? This includes any government, private or employer-paid insurance. *Check one only.*

- ☐ Yes
- ☐ No
- ☐ Don't know
- ☐ REFUSED (option is hidden on Voxco)

1.16 Since the start of the COVID-19 pandemic, what types of places have you lived in? *Select all that apply.*

- ☐ Own apartment or house
- ☐ Family or friend's place
- ☐ Couch surfing, multiple residences
- ☐ Hotel or motel room
- ☐ Rooming or boarding house
- ☐ Shelter or hostel
- ☐ Transition house or halfway house
- ☐ Psychiatric institution or drug treatment facility, like detox or rehab, or HIV supported housing
- ☐ Public place, like a street, park or stairwell
- ☐ Correctional facility, like jail or prison
- ☐ Other
- ☐ REFUSED (option is hidden on Voxco)

Mental health and wellness

The following questions are about your experience during the COVID-19 pandemic. The next few questions are about mental health and wellness.

2.1 In general, how would you describe your mental health? *Check one only.*

- ☐ Excellent
- ☐ Very good
- ☐ Good
- ☐ Fair
- ☐ Poor
- ☐ REFUSED (option is hidden on Voxco)

2.2 Compared to before the COVID-19 pandemic started, how would you say your mental health is now? Would you say…? *Check one only.*

- ☐ Much better now
- ☐ Somewhat better now
- ☐ About the same
- ☐ Somewhat worse now
- ☐ Much worse now
- ☐ REFUSED (option is hidden on Voxco)

2.3 Since the start of the COVID-19 pandemic, did you access, consider accessing, or want to access mental health and wellness services (e.g., mental health care providers, community supports, faith-based and spiritual care supports, etc.)? *Check one only.*

- ☐ Yes
- ☐ No → *Go to question 3.1*
- ☐ REFUSED (option is hidden on Voxco)

2.4 Since the start of the COVID-19 pandemic, were you ever not able to access any of these mental health and wellness services? *Check one only.*

- ☐ I was not able to access services
- ☐ I was sometimes not able to access services and sometimes able to access services
- ☐ I was always able to access services → *Go to question 3.1*
- ☐ REFUSED (option is hidden on Voxco)

2.5 Since the start of the COVID-19 pandemic, why were you <u>not able</u> to access mental health and wellness services? *Select all that apply.*

- ☐ Difficulty getting a referral
- ☐ Difficulty getting an appointment
- ☐ Difficulty contacting a doctor or nurse to get information or advice
- ☐ Waited too long between booking an appointment and visit
- ☐ Waited too long to get healthcare service
  i.e., in-office waiting
- ☐ The service was not available at time required
  i.e., reduced hours of operation or service was closed
- ☐ Difficulty accessing service because of COVID-19 related public health measures
  e.g., experiencing symptoms, self-isolating, physical distancing, entry screening procedures, childcare or school closures
- ☐ Fear of, or concern about exposure to someone with COVID-19
- ☐ Fear of, concern about or experienced stigma, discrimination, or violence
- ☐ Fear of, concern about or experienced anti-Black racism
- ☐ Difficulty accessing remote services
  e.g., not comfortable using remote services, lack of access to technology or internet
- ☐ Culturally safe and responsive services were not available
- ☐ Transportation problems
- ☐ Language problem
- ☐ Cost
- ☐ Other
- ☐ REFUSED (option is hidden on Voxco)

Financial state, housing stability, and food security

3.1 Before the COVID-19 pandemic, what was your work situation? *Select all that apply.*

- ☐ Employed or self-employed full time
- ☐ Employed or self-employed part time
- ☐ Volunteering
- ☐ Unemployed
- ☐ A full or part time student
- ☐ Not working due to a disability
- ☐ Looking after children or other family members
- ☐ Retired
- ☐ Other
- ☐ Don't know
- ☐ REFUSED (option is hidden on Voxco)

3.2 Since the start of the COVID-19 pandemic, did you experience changes in your work situation? *Check one only.*

- ☐ Little to no change → *Go to question 3.4*
- ☐ Reduced hours and/or pay
- ☐ Increased hours and/or pay → *Go to question 3.4*
- ☐ Had to stop working
- ☐ REFUSED (option is hidden on Voxco)

3.3 What was the <u>main reason</u> you had to limit your work or stop working during the COVID-19 pandemic? *Check one only.*

- ☐ Planned absence not related to COVID-19
  e.g., vacation, work schedule, maternity or parental leave, seasonal job or business
- ☐ Unplanned absence not related to COVID-19
  e.g., illness or disability other than COVID-19, caring for children or elder relative for non-COVID-19 reasons, labour dispute (strike or lockout)
- ☐ Business closure or layoff related to COVID-19
- ☐ Personal circumstances related to COVID-19
  e.g., personal safety, own or household member's exposure, self-isolation after recent travel, taking care of children due to school and/or daycare closures
- ☐ Other
- ☐ REFUSED (option is hidden on Voxco)

3.4 Which of the following best describes the impact of COVID-19 pandemic on your ability to pay your bills for essential needs, such as rent or mortgage payments, utilities, and groceries? *Check one only.*

- ☐ Major impact
- ☐ Moderate impact
- ☐ Minor impact
- ☐ No impact
- ☐ Too soon to tell
- ☐ REFUSED (option is hidden on Voxco)

3.5 Since the start of the COVID-19 pandemic, did you receive any of the following types of Employment Insurance benefits or the Canada Emergency Response Benefit? *Select all that apply.*

- ☐ Regular Employment Insurance benefits
- ☐ Sickness
- ☐ Caregiving or compassionate care
- ☐ Work-sharing
- ☐ Other Employment Insurance benefit
- ☐ Canada Emergency Response Benefit (CERB)
- ☐ Canada Emergency Student Benefit (CESB)
- ☐ Did not apply for any benefits
- ☐ Did not qualify for any benefits
- ☐ REFUSED (option is hidden on Voxco)

3.6 Since the start of the COVID-19 pandemic, have you received relief or payment deferrals for any of the following financial obligations? *Check one only.*

	I have these payments and I received relief or payment deferrals	I have these payments and I needed relief or payment deferrals but didn't receive them	I have these payments but I didn't need relief or payment deferrals	I do not have these payments	REFUSED (option is hidden on Voxco)
Rent or mortgage payments	☐	☐	☐	☐	☐
Car payments	☐	☐	☐	☐	☐
Household bills	☐	☐	☐	☐	☐

3.7 Since the start of the COVID-19 pandemic, indicate if the following statements were true…
Check one only.

	Often true	Sometimes true	Never true	REFUSED (option is hidden on Voxco)
The food that you or other household members bought just didn't last, and there wasn't any money to get more.	☐	☐	☐	☐
You or other household members couldn't afford to eat balanced meals.	☐	☐	☐	☐
You ate less than you felt you should because there wasn't enough money to buy food.	☐	☐	☐	☐
Others in your household ate less than you felt they should because there wasn't enough money to buy food.	☐	☐	☐	☐
You or other household members accessed food or meals, at no cost to you, from a community organization.	☐	☐	☐	☐

Domestic violence

4.1 Since the start of the COVID-19 pandemic, which one of the following BEST describes your living arrangement? *Check one only.*

☐ Living alone
☐ Living with family
e.g., parents, siblings, spouse or partner and/or children, in-laws, etc.
☐ Living with roommate(s) or friend(s)
☐ Living in a shelter
☐ I am homeless
☐ Prefer not to answer
☐ REFUSED (option is hidden on Voxco)

4.2 Thinking about <u>the year before</u> the start of the COVID-19 pandemic, how safe did you feel where you live? *Check one only.*

☐ Very safe
☐ Somewhat safe
☐ Not very safe
☐ Not safe at all
☐ Don't know
☐ REFUSED (option is hidden on Voxco)

4.3 Since the start of the COVID-19 pandemic, do you feel more or less safe where you live? *Check one only.*

☐ Less safe
☐ The same
☐ More safe
☐ REFUSED (option is hidden on Voxco)

4.4 The following questions ask about experiences where you live. Since the start of the COVID-19 pandemic, how often did someone… *Check one only.*

	Less often	More often	No change	I've never experienced this or this does not apply to me	Prefer not to answer	REFUSED (option is hidden on Voxco)
Yell at you or said things to you that made you feel bad about yourself, embarrassed you in front of	☐	☐	☐	☐	☐	☐

others, or frightened you?						
Do things like push, grab, hit, slap, kick, or throw things at you during an argument or because they were angry with you?	☐	☐	☐	☐	☐	☐
Be more sexually aggressive towards you?	☐	☐	☐	☐	☐	☐
Yell at someone you live with?	☐	☐	☐	☐	☐	☐
Do things like push, grab, hit, slap, kick, or throw things at someone you live with?	☐	☐	☐	☐	☐	☐
Control how money was spent in your household including limiting your access or withholding funds from you?	☐	☐	☐	☐	☐	☐

Racism, stigma and discrimination

5.1 Thinking about <u>the year before</u> the start of the COVID-19 pandemic, did you experience discrimination when accessing healthcare services because of your race or ethnicity or skin colour (including anti-Black racism), gender, sexual orientation, use of substances, economic status, (dis)ability, or other identity? *Check one only.*

- ☐ Often
- ☐ Sometimes
- ☐ Rarely
- ☐ Never
- ☐ Don't know
- ☐ I did not access healthcare services
- ☐ REFUSED (option is hidden on Voxco)

5.2 Since the start of the COVID-19 pandemic, has the frequency of your experiences of discrimination increased, decreased, or remained about the same when you accessed healthcare services? *Check one only.*

- ☐ Strong decrease
- ☐ Slight decrease
- ☐ No change
- ☐ Slight increase
- ☐ Strong increase
- ☐ Don't know
- ☐ I did not access healthcare services → *Go to question 6.1*
- ☐ REFUSED (option is hidden on Voxco)

5.3 Since the start of the COVID-19 pandemic, when you accessed healthcare services, did you experience more or less discrimination based on any of the following? *Check one only.*

	Increase	Decrease	No change	Did not experience	REFUSED (option is hidden on Voxco)
Race or ethnicity or skin colour, including anti-Black racism	☐	☐	☐	☐	☐
Gender	☐	☐	☐	☐	☐
Sexual orientation	☐	☐	☐	☐	☐
Use of substances	☐	☐	☐	☐	☐
Economic status	☐	☐	☐	☐	☐
(Dis)ability	☐	☐	☐	☐	☐
Age	☐	☐	☐	☐	☐

Substance use and harm reduction services

The next set of questions is about your use of substances, including legal substances like alcohol and cannabis, and non-legal substances like cocaine, methamphetamine, hallucinogens, heroin and other non-medical use of opioids, etc.

6.1 Thinking about the year before the start of the COVID-19 pandemic, did you use any substances, including alcohol, cannabis or other drugs? *Check one only.*

- ☐ Yes
- ☐ No
- ☐ Prefer not to answer
- ☐ REFUSED (option is hidden on Voxco)

6.2 Since the start of the COVID-19 pandemic, generally did your consumption of the following substances change? *Check one only.*

	Increase	Decrease	No change	I do not use this	REFUSED (option is hidden on Voxco)	Prefer not to answer
Alcohol	☐	☐	☐	☐	☐	☐
Cannabis	☐	☐	☐	☐	☐	☐
Cocaine or crack	☐	☐	☐	☐	☐	☐
Speed, methamphetamine or crystal meth	☐	☐	☐	☐	☐	☐
Hallucinogens	☐	☐	☐	☐	☐	☐
Ecstasy	☐	☐	☐	☐	☐	☐
Heroin, fentanyl or other non-medical opioids	☐	☐	☐	☐	☐	☐
Other substances	☐	☐	☐	☐	☐	☐

→ If all responses "I do not use this", go to question 7.1

→ If all refused, go to question 6.3

6.3 Since the start of the COVID-19 pandemic, generally did you experience changes in any of the following? *Check one only.*

	Increase	Decrease	No change	Not applicable	REFUSED (option is hidden on Voxco)	Prefer not to answer
Shared used equipment such as needles or syringes, pipes, tourniquets, swabs, cookers	☐	☐	☐	☐	☐	☐
Used substances I do not usually use	☐	☐	☐	☐	☐	☐
Had withdrawal symptoms	☐	☐	☐	☐	☐	☐
Was unable to get the substances I use	☐	☐	☐	☐	☐	☐
Used alone	☐	☐	☐	☐	☐	☐
Worried about overdosing	☐	☐	☐	☐	☐	☐
Had different triggers for using	☐	☐	☐	☐	☐	☐

6.4 Since the start of the COVID-19 pandemic, did you access, consider accessing or want to access substance-related services (needle or syringe distribution, on-site consumption, drug checking, naloxone training and provision, etc.)? *Check one only.*

☐ Yes
☐ No → *Go to question 6.7*
☐ REFUSED (option is hidden on Voxco)

→ *If refused, go to question 6.5*

6.5 Since the start of the COVID-19 pandemic, which substance-related services did you access, consider accessing or want to access? *Check one only.*

	Always able to access	Sometimes able to access	Wanted or tried to, but was not able to access	Did not try to access	REFUSED (option is hidden on Voxco)
Needle and syringe distribution programs	☐	☐	☐	☐	☐
Drop-in centres for people who use drugs	☐	☐	☐	☐	☐
Drug consumption rooms	☐	☐	☐	☐	☐
Drug checking services	☐	☐	☐	☐	☐
Outreach services	☐	☐	☐	☐	☐
Naloxone training	☐	☐	☐	☐	☐
Educational resources on safer drug use	☐	☐	☐	☐	☐
Delivery service for safe drug supplies	☐	☐	☐	☐	☐
Community Services (e.g., Peer support services)	☐	☐	☐	☐	☐

→ If all responses "Always able to access", go to question 6.7

→ If all refused, go to question 6.7

6.6 Since the start of the COVID-19 pandemic, why were you not able to access substance-related services? *Select all that apply.*

- ☐ Difficulty getting a referral
- ☐ Difficulty getting an appointment
- ☐ Difficulty contacting a doctor or nurse to get information or advice
- ☐ Waited too long between booking an appointment and visit
- ☐ Waited too long to get healthcare service
  i.e., in-office waiting
- ☐ The service was not available at time required
  i.e., reduced hours of operation or service was closed
- ☐ Difficulty accessing service because of COVID-19 related public health measures
  e.g., experiencing symptoms, self-isolating, physical distancing, entry screening procedures, childcare or school closures
- ☐ Fear of, or concern about exposure to someone with COVID-19
- ☐ Fear of, concern about or experienced stigma, discrimination, or violence
- ☐ Fear of, concern about or experienced anti-Black racism
- ☐ Difficulty accessing remote services
  e.g., not comfortable using remote services, lack of access to technology or internet
- ☐ Culturally safe and responsive services were not available
- ☐ Transportation problems
- ☐ Language problem
- ☐ Cost
- ☐ Other
- ☐ REFUSED (option is hidden on Voxco)

6.7 Since the start of the COVID-19 pandemic, did you access, consider accessing or want to access substance-related treatment services (counselling, opioid substitution treatment (OST), inpatient services, community-based programs and services, etc.)? *Check one only.*

- ☐ Yes
- ☐ No → *Go to question 7.1*
- ☐ REFUSED (option is hidden on Voxco)

→ *If refused, go to question 6.8*

6.8 Since the start of the COVID-19 pandemic, which substance-related treatment services did you access, consider accessing or want to access? *Check one only.*

	Always able to access	Sometimes able to access	Wanted or tried to, but was not able to access	Did not try to access	REFUSED (option is hidden on Voxco)
Outpatient counselling and psychosocial treatment	☐	☐	☐	☐	☐
Opioid substitution treatment (OST) in specialized outpatient treatment centres	☐	☐	☐	☐	☐
OST in non-specialized outpatient treatment centres (e.g., provided by a family doctor or nurse practitioner)	☐	☐	☐	☐	☐
Drug treatment in primary healthcare settings (e.g., provided by a family doctor or nurse practitioner)	☐	☐	☐	☐	☐
Drug treatment in outpatient mental health care centres	☐	☐	☐	☐	☐
Drug treatment in hospital-based residential settings (psychiatric hospitals)	☐	☐	☐	☐	☐
Drug treatment in non-hospital based residential settings (therapeutic communities)	☐	☐	☐	☐	☐
Indigenous health or healing services	☐	☐	☐	☐	☐
Overdose prevention and response	☐	☐	☐	☐	☐
Community support services (e.g., Peer support services, linkage to safe injection sites and treatment)	☐	☐	☐	☐	☐

→ If all responses "Always able to access", go to question 7.1

→ If all refused, go to question 7.1

6.9 Since the start of the COVID-19 pandemic, why were you <u>not able</u> to access substance-related treatment services? *Select all that apply.*

- ☐ Difficulty getting a referral
- ☐ Difficulty getting an appointment
- ☐ Difficulty contacting a doctor or nurse to get information or advice
- ☐ Waited too long between booking an appointment and visit
- ☐ Waited too long to get healthcare service
  i.e., in-office waiting
- ☐ The service was not available at time required
  i.e., reduced hours of operation or service was closed
- ☐ Difficulty accessing service because of COVID-19 related public health measures
  e.g., experiencing symptoms, self-isolating, physical distancing, entry screening procedures, childcare or school closures
- ☐ Fear of, or concern about exposure to someone with COVID-19
- ☐ Fear of, concern about or experienced stigma, discrimination, or violence
- ☐ Fear of, concern about or experienced anti-Black racism
- ☐ Difficulty accessing remote services
  e.g., not comfortable using remote services, lack of access to technology or internet
- ☐ Culturally safe and responsive services were not available
- ☐ Transportation problems
- ☐ Language problem
- ☐ Cost
- ☐ Other
- ☐ REFUSED (option is hidden on Voxco)

STBBI prevention, testing and treatment services

7.1 Since the start of the COVID-19 pandemic, did you access, consider accessing or want to access sexually transmitted and blood-borne infection (STBBI) prevention, testing and treatment services (STBBI testing and treatment, oral HIV pre-exposure prophylaxis (PrEP) or post-exposure prophylaxis (PEP) provision, condom and/or dental dam provision, etc.)?
Check one only.

- ☐ Yes
- ☐ No → *Go to question 7.4*
- ☐ REFUSED (option is hidden on Voxco)
  → *If refused, go to question 7.2*

7.2. Since the start of the COVID-19 pandemic, which STBBI prevention, testing and treatment services did you access, consider accessing or want to access? *Check one only.*

	Always able to access	Sometimes able to access	Wanted or tried to, but was not able to access	Did not try to access	REFUSED (option is hidden on Voxco)
HIV testing	☐	☐	☐	☐	☐
Hepatitis C testing	☐	☐	☐	☐	☐
Other sexually transmitted infection (STI) testing	☐	☐	☐	☐	☐
PrEP and/or PEP	☐	☐	☐	☐	☐
Condom and/or dental dam	☐	☐	☐	☐	☐
Resources about safer sex (postcard, pamphlets, etc.)	☐	☐	☐	☐	☐
STBBI information and education including outreach events (e.g., health fairs, festivals, community events, etc.)	☐	☐	☐	☐	☐
Mental health counselling referral	☐	☐	☐	☐	☐
Pre and post HIV test counselling	☐	☐	☐	☐	☐
Indigenous health or healing services	☐	☐	☐	☐	☐
Community services (e.g., Peer support services)	☐	☐	☐	☐	☐
Interpreter and/or peer health service navigator	☐	☐	☐	☐	☐

→ *If all responses "Always able to access", go to question 7.4*

→ *If all refused, go to question 7.4*

7.3 Since the start of the COVID-19 pandemic, why were you <u>not able</u> to access STBBI prevention, testing and treatment services? *Select all that apply.*

- ☐ Difficulty getting a referral
- ☐ Difficulty getting an appointment
- ☐ Difficulty contacting a doctor or nurse to get information or advice
- ☐ Waited too long between booking an appointment and visit
- ☐ Waited too long to get healthcare service
  i.e., in-office waiting
- ☐ The service was not available at time required
  i.e., reduced hours of operation or service was closed
- ☐ Difficulty accessing service because of COVID-19 related public health measures
  e.g., experiencing symptoms, self-isolating, physical distancing, entry screening procedures, childcare or school closures
- ☐ Fear of, or concern about exposure to someone with COVID-19
- ☐ Fear of, concern about or experienced stigma, discrimination, or violence
- ☐ Fear of, concern about or experienced anti-Black racism
- ☐ Difficulty accessing remote services
  e.g., not comfortable using remote services, lack of access to technology or internet
- ☐ Culturally safe and responsive services were not available
- ☐ Transportation problems
- ☐ Language problem
- ☐ Cost
- ☐ Other
- ☐ REFUSED (option is hidden on Voxco)

7.4 Are you currently living with HIV? *Check one only.*

- ☐ Yes
- ☐ No → *Go to question 7.8*
- ☐ Don't know → *Go to question 7.8*
- ☐ Prefer not to answer → *Go to question 7.8*
- ☐ REFUSED (option is hidden on Voxco)

→ *If refused, go to question 7.8*

7.5 Thinking about <u>the year before</u> the start of the COVID-19 pandemic, did you have a clinic or HIV care provider that you went to for your HIV care? *Check one only.*

- ☐ Yes
- ☐ No
- ☐ I received my HIV diagnosis during the COVID-19 pandemic
- ☐ REFUSED (option is hidden on Voxco)

7.6 Since the start of the COVID-19 pandemic, did you experience any challenges accessing an HIV care provider or clinic? *Check one only.*

☐ Yes
☐ No → *Go to question 7.8*
☐ I didn't try to access an HIV care provider or clinic → *Go to question 7.8*
☐ REFUSED (option is hidden on Voxco)

7.7 Since the start of the COVID-19 pandemic, why did you experience challenges accessing HIV care? *Select all that apply.*

☐ Difficulty getting a referral
☐ Difficulty getting an appointment
☐ Difficulty contacting a doctor or nurse to get information or advice
☐ Waited too long between booking an appointment and visit
☐ Waited too long to get healthcare service
i.e., in-office waiting
☐ The service was not available at time required
i.e., reduced hours of operation or service was closed
☐ Difficulty accessing service because of COVID-19 related public health measures
e.g., experiencing symptoms, self-isolating, physical distancing, entry screening procedures, childcare or school closures
☐ Fear of, or concern about exposure to someone with COVID-19
☐ Fear of, concern about or experienced stigma, discrimination, or violence
☐ Fear of, concern about or experienced anti-Black racism
☐ Difficulty accessing remote services
e.g., not comfortable using remote services, lack of access to technology or internet
☐ Culturally safe and responsive services were not available
☐ Transportation problems
☐ Language problem
☐ Cost
☐ Other
☐ REFUSED (option is hidden on Voxco)

7.8 Have you ever been told that you have hepatitis C? *Check one only.*

☐ Yes
☐ No → *Go to question 7.13*
☐ Prefer not to answer → *Go to question 7.13*
☐ REFUSED (option is hidden on Voxco)

→ *If refused, go to question 7.13*

7.9 Do you currently have hepatitis C? *Check one only.*

☐ Yes – I have hepatitis C
☐ No – I cleared the virus spontaneously → *Go to question 7.13*
☐ No – I cleared the virus with treatment → *Go to question 7.13*
☐ I don't know → *Go to question 7.13*
☐ Prefer not to answer → *Go to question 7.13*
☐ REFUSED (option is hidden on Voxco)

→ *If refused, go to question 7.13*

7.10 Thinking about <u>the year before</u> the start of the COVID-19 pandemic, did you have a clinic or hepatitis C care provider that you went to for your hepatitis C care? *Check one only.*

☐ Yes
☐ No
☐ I received my hepatitis C diagnosis during the COVID-19 pandemic
☐ REFUSED (option is hidden on Voxco)

7.11 Since the start of the COVID-19 pandemic, did you experience any challenges accessing a hepatitis C care provider or clinic? *Check one only.*

☐ Yes
☐ No → *Go to question 7.13*
☐ I didn't try to access a hepatitis C care provider or clinic → *Go to question 7.13*
☐ REFUSED (option is hidden on Voxco)

→ *If refused, go to question 7.12*

7.12 Since the start of the COVID-19 pandemic, why did you experience challenges accessing hepatitis C care? *Select all that apply.*

☐ Difficulty getting a referral
☐ Difficulty getting an appointment
☐ Difficulty contacting a doctor or nurse to get information or advice
☐ Waited too long between booking an appointment and visit
☐ Waited too long to get healthcare service
i.e., in-office waiting
☐ The service was not available at time required
i.e., reduced hours of operation or service was closed
☐ Difficulty accessing service because of COVID-19 related public health measures
e.g., experiencing symptoms, self-isolating, physical distancing, entry screening procedures, childcare or school closures
☐ Fear of, or concern about exposure to someone with COVID-19
☐ Fear of, concern about or experienced stigma, discrimination, or violence
☐ Fear of, concern about or experienced anti-Black racism
☐ Difficulty accessing remote services
e.g., not comfortable using remote services, lack of access to technology or internet
☐ Culturally safe and responsive services were not available
☐ Transportation problems
☐ Language problem
☐ Cost
☐ Other
☐ REFUSED (option is hidden on Voxco)

7.13 Is there anything else you'd like to share about your experience during the COVID-19 pandemic? For example, how you were able to access services in ways that were different from before the COVID-19 pandemic or what worked to keep services available and accessible.

____

____

____

7.14 Do you give permission for words and statements from your response to be quoted anonymously in reports prepared from this survey?

☐ Yes
☐ No
☐ REFUSED (option is hidden on Voxco)

Ending message #1b:

This is the end of the survey.

Hit the **Submit** button to finish the survey.

Back Submit

Ending message #2b:

Thank you for taking the time to fill out this important survey.

If you think you need mental health support after completing the survey, help and resources are available to you at this website: Mental health support: Get help

If you need mental health support specific to African, Caribbean and Black communities, help and resources are available to you at this website: Mental Health Resources for Black communities in Canada

Find more information on taking care of your mental and physical health during the COVID-19 pandemic, please visit: COVID-19: Taking care of your mental and physical health during the COVID-19 pandemic

For more information on COVID-19 for Canadians including measures to reduce COVID-19 in your community, please visit: Coronavirus disease (COVID-19)

Stay safe!
